# Financial Socialization, Childhood Experiences and Financial Well-Being: The Mediating Role of Locus of Control

**DOI:** 10.3389/fpsyg.2020.02162

**Published:** 2020-09-29

**Authors:** Saif Ullah, Kong Yusheng

**Affiliations:** ^1^School of Finance and Economics, Jiangsu University, Zhenjiang, China; ^2^Department of Management Sciences, National University of Modern Languages, Islamabad, Pakistan

**Keywords:** financial satisfaction, financial decision making, financial well-being, financial socialization, locus of control, early childhood consumer experiences

## Abstract

The present study evaluates an empirical model of financial well-being (FWB) based on early childhood consumer experiences (ECCE) and financial socialization (FS). FWB is the overall satisfaction with one’s current financial situation, and that plays a vital role in the overall success and helps to overcome psychological health issues among adults. The results of the study suggested that ECCE has a significant direct effect on the FWB among adults. Moreover, FS agents such as peers harm the financial well-being of the adults. The results also revealed that financial locus of control (LOC) mediates the relationship between FS agents such as parents, teachers, and FWB. Surprisingly, FS agent peers and ECCE do not affect the FWB of adults when LOC works as a mediator. Possibly, when adults socialize more with parents and teachers, they often disregard the role of peers. Adults’ belief and confidence in their skills are vital in explaining the above relationships. Educationists and practitioners should focus on improving discussions with parents and teachers about the financial matters that result in improvements in financial well-being. The present study also provides theoretical and practical implications for adults, parents, and policymakers.

## Introduction

For decades, researchers, financial counselors, financial planners, educationists, economists have been interested in investigating the methods to improve the quality of life by exploring the potential sources of well-being (Porter and Garman, 1993; Muffels and Headey, 2009; Shim et al., 2009a; Serido et al., 2010; Brüggen et al., 2017; Abrantes-Braga and Veludo-de-Oliveira, 2019). Well-being is also considered vital as it overcomes interpersonal, family, and health problems (McLellan, 2017). Despite the importance of well-being, there is no consensus over the definition, measures, and factors affecting well-being (Rea, 2017). The individual’s well-being consists of social, psychological, physical, and financial well-being (Zemtsov and Osipova, 2016). In addition to this, Netemeyer et al. (2018) depicted that financial well-being is the fundamental predictor of the well-being of the adults.

Financial Well-being (FWB) is a relatively new concept in personal financial management. Although there is a growing interest of scholars on the FWB, there is no consensus over the definition, measures, and factors affecting financial well-being (Rea, 2017). According to Shim et al. (2009a), FWB is the overall satisfaction with one’s financial situation. While Drever et al. (2015) explained FWB is the ability to have control over one’s daily and monthly finances, the capacity to handle financial uncertainties, meet financial goals, and have the financial freedom to make choices that allow one to enjoy life. Definition by Shim et al. (2009a) explained FWB as the outcomes of financial management, while Drever et al. (2015) explained it as capabilities to manage finances. FWB can be objective or subjective. Danes and Yang (2014) explained that objective measures of FWB are income adequacy, net worth, e.g., Shim et al. (2009a) explained overall satisfaction as a subjective indicator of FWB. By following Shim et al. (2009a), we have considered FWB as satisfaction with one’s financial situation.

Achieving financial well-being (FWB) is essential for adults as it can affect physical, psychological, and social health, which results in unsatisfactory job performance, inability to concentrate, lower productivity, and absenteeism. Nowadays, the majority of young adults encounter considerable threats to their FWB, while most of the adults anticipate to manage these threats and to attain financial well-being but fail to do so. The resources and opportunities that individuals have played a vital role in the success or failure of FWB. Regardless of the differences in resources and opportunities that lead to differences in financial well-being, certain behaviors can enrich FWB across individuals. These behaviors facilitate in making informed financial decisions through better future planning and managing resources effectively. The prevailing literature advocates that a variety of socio-economic characteristics, as well as a diverse set of personal attributes, guide about these behaviors (Gutter and Copur, 2011; Sabri, 2011; Danes and Yang, 2014; Strömbäck et al., 2017; Fan et al., 2018).

In determining the financial well-being, studies have identified different contributing factors, i.e., proficiency in financial skills (Kim and Chatterjee, 2013; Xiao et al., 2013); marital status, physical health, income (Cox et al., 2009); interaction with parents, attitudes toward knowledge (Bornstein, 2005; Serido et al., 2010; Mohamed, 2017); financial knowledge, interaction with socialization agents and early childhood consumer experiences (Sabri and MacDonald, 2010; Sabri, 2011); positive financial behaviors (Shim et al., 2009a); parent-child communication and parents expectations (Bornstein, 2005; Serido et al., 2010; Drever et al., 2015; Gudmunson et al., 2015). The present study is an attempt to answer the question of what are the relevant factors that contribute to an individual’s financial well-being.

Financial Socialization (FS) can have an impact on the FWB. FS, as explained by Danes (1994), is the process of learning and advancing values, knowledge, norms, standards, attitudes, and behaviors which promote well-being, financial viability among the individuals. Although FS is vital for understanding the financial behaviors of young adults, literature is silent about the FS process from the perceptions of young adults. Most often, due to the lack of observational and qualitative data, the understanding of these behaviors is ignored (Rea, 2017). Previous studies suggest that children learn financial socialization processes at a young age from their families, thus influencing their future financial behaviors and FWB, e.g., (Shim et al., 2009a; Gudmunson and Danes, 2011; Kim and Chatterjee, 2013; Gudmunson et al., 2015). Based on the literature, we have included financial socialization as a potential indicator of financial well-being.

The empirical research on FWB also highlighted the importance of Early Childhood Consumer Experiences (ECCE) in explaining financial well-being. ECCE is included in the model because of its relevance, as suggested by the theory of life span (Baltes, 1987). The theory of life-span offered a viewpoint to examine both the constancy and the variations in the behaviors at the different stages of an individual’s life span. Results of developmental studies have demonstrated that ECCE has a positive influence on the financial management skills of the adults (Sabri and MacDonald, 2010). The present study is an attempt to examine the effect of ECCE on the financial well-being of the adults.

For the last few decades, the researchers are trying to employ Locus of Control (LOC) in the field of personal finance and economics (Prawitz and Cohart, 2016). LOC is the belief of individuals that they are in control of their future. LOC has an impact on the behaviors as well as financial and non-financial preferences of the individuals (Onkvisit and Shaw, 1987; Shim et al., 2009a; Prawitz and Cohart, 2016). LOC is also associated with the financial satisfaction of individuals (Sumarwan and Hira, 1993). In this way, we can say that LOC can influence the FWB of adults. Although locus of control is not a new phenomenon, only a few studies are available on the locus of control (Rotter, 1966; Ajzen, 2002; Norvilitis et al., 2003; Griffin, 2014; Furnham and Cheng, 2017). The underlying intention of the present study is to examine locus of control and its mediating effect between FS, ECCE, and FWB.

This study attempts to add to our existing knowledge about personal attributes as well as the financial socialization process that improves an individual’s FWB. To do so, we have offered a model of FS and early childhood consumer experience on the financial locus of control, and their association with financial well-being. Although developmental studies have observed these factors (Shim et al., 2009b; Gerrans et al., 2013; Rea, 2017; Ponchio et al., 2019; Sabri and Anthony, 2019), they have used other sets of variables to study financial well-being.

The present study is conducted on the adults of Pakistan. Pakistan is included in the six most populous countries in the world. In addition to this, Pakistan is also included in the countries where there is the highest level of population growth rate. The majority (around 60% are of 30 year age) of the population of Pakistan are adults. However, due to instability in the political system, unfortunate financial situation of the country, poor health and educational system, increasing unemployment rate, and high population growth rate, the country is unable to take benefit this youth. A recent report published by the UNDP shows that Pakistan is ranked in the 152nd position out of 189 countries in the Human Development Index (HDI). The risk of mental health problems like depression is higher among the unemployed than among the employed. All this is related to the quality of life, and one way to improve the quality of life is to improve the financial well-being of the adults. That is why the present study is necessary for Pakistan as it will guide about factors that are related to the financial well-being of the adults that will ultimately enhance the quality of life of the adults.

## Theoretical Background and Hypothesis

### Financial Socialization (FS) and Financial Well-Being (FWB)

Financial Socialization (FS) is a process of learning and advancing values, knowledge, norms, standards, attitudes, and behaviors that promote financial viability and individual well-being (Danes, 1994). FS is not only related to managing money; it includes the development of standards, values, norms, and attitudes that will hinder or support the development of financial capability among individuals as well as improve financial well-being. Although in psychology, attitude development is an emerging area for researchers, only a few researchers have focused on the development of financial attitudes in children. It is an established phenomenon that financial behaviors that individuals follow over their adulthood were the result of financial attitudes that they have learned in their childhood (Drever et al., 2015).

Adults learn about consumer knowledge and behaviors in their childhood through the interaction with socialization agents such as parents, siblings, other family members, peers, religion, schools and then practice these behaviors and knowledge in their adulthood years (Gudmunson and Danes, 2011; Gutter and Copur, 2011; Drever et al., 2015). Recent literature has established that many of the FS outcomes (e.g., positive financial behaviors, FWB) of young adults are embedded in the FS processes experienced in childhood (e.g., Shim et al., 2009a; Drever et al., 2015; Gudmunson et al., 2015). Recently, Jorgensen et al. (2016b) studied the impact of FS on financial decision making and concluded that individuals who have more opportunities to interact and observe financial socialization agents perform better in making financial decisions. Rea et al. (2019) also confirmed the findings of FS theory that financially socialized individuals perform better in making sound financial decisions in their adulthood and achieve financial well-being.

Based on the literature, the hypothesis of the study are:

**H1:** *FS will be associated with FWB among young adults.*
**H1a:** *Parent as a financial socialization agent affects the FWB of young adults.*
**H1b:** *Peers as the financial socialization agent affect the FWB of young adults.*
**H1c:** *Teachers play a significant role in explaining the FWB of adults.*

### Early Childhood Consumer Experiences (ECCE)

Socialization begins in childhood and continues to some extent, throughout one’s lifetime (Danes, 1994). Baltes (1987) proposed the theory of life span. It offers a viewpoint to examine the steadiness and variations in the behaviors at the different stages of an individual’s life span. From the perspective of the theory of life span, throughout at different stages of an individual’s life span, different developmental tasks may become vital for a while. When individuals attain maturity, these tasks are affected by the social-economic factors, and turn out to be complicated, and formulate the foundations which provide the basis for the subsequent behaviors. This theory states that early childhood consumer experiences have a significant impact on the financial behaviors of individuals.

Danes and Yang (2014) also noted that, as children mature and form their own families, they try to follow financial behavior patterns that they have learned during their childhood. Another study by Sabri and MacDonald (2010) revealed that those students who had ECCE were more likely to engage in active financial behavior and less likely to report financial problems (Sabri and MacDonald, 2010). Falahati and Sabri (2015) also concluded their study that childhood consumer experiences are significant determinants of financial strain among adults. Hence, we can conclude that childhood consumer experiences help in making sound financial decisions and control financial strain that improves the financial well-being of the adults.

Based on the theory of life span and recent literature, the second hypothesis of the study is:

H_2_: ECCE affect the FWB of the individuals.

### Mediating Role of Financial Locus of Control (LOC)

Research on consumer socialization suggests that adults develop their knowledge and behaviors in their childhood through the interaction with socialization agents such as parents, other family members, peers, religion, teachers, schools and practice these behaviors and knowledge in their adulthood years (Churchill and Moschis, 1979; Gutter et al., 2010; Gudmunson and Danes, 2011; Drever et al., 2015). The interaction with financial socialization agents and ECCE provides confidence to the individuals about managing their financials (Glenn, 2018). This confidence in managing financials is related to financial locus of control (LOC). From this, we can say that the financial locus of control is the outcome of financial socialization (parents, peers and teachers) and early childhood consumer experiences.

Locus of control is the degree to which individuals believe they are in control of their future. LOC has an impact on the behaviors as well as financial and non-financial preferences of individuals (Jorgensen et al., 2016a; Kempson and Poppe, 2018; Kesavayuth et al., 2018). LOC is also related to financial satisfaction (Sumarwan and Hira, 1993). LOC has two continuums: internal and external. Individuals having internal LOC believe that they can influence consequences in their lives, or their actions affect outcomes about later stages of life, whereas individuals with external LOC consider that outcomes are the result of luck or destiny and are outside of personal control. Although locus of control is not a new phenomenon, only a few studies are available on the financial locus of control (Jorgensen et al., 2016a; Kempson and Poppe, 2018; Kesavayuth et al., 2018). Jorgensen et al. (2016b) argued that internal locus of control is more relevant in the studies related to personal financial management. Thus by following relevant literature, the researcher has used only internal LOC.

Furthermore, their financial well-being is affected by the association of these behaviors and the financial socialization process.

**H3:** *Financial LOC mediates the relationship between FS (Teachers, Parents, and Peers) and FWB.*
**H4:** *Financial LOC mediates the relationship between Early Childhood consumer experiences and FWB.*

### Theoretical Framework

**FIGURE 1:**
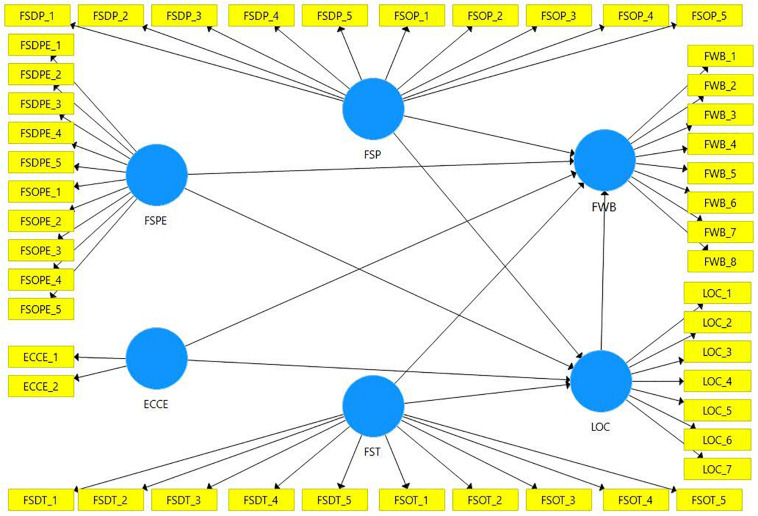
Theoretical framework.

Based on the relevant literature and hypotheses, Figure 1 shows the theoretical framework followed in the present study. Figure 1 shows that Financial Socialization through Parents, Peers, and Teachers is is an independent variable along with ECCE. FWB is a dependent variable. Locus of control tries to explain the relationship between Financial Socialization agents, Early childhood consumer experiences, and Financial well-being. The items used to measure the variables are shown in the yellow boxes, while blue cicles are used to show variables.

## Materials and Methods

It consists of various sections including (a) Population, sampling, and collection of data, (b) Questionnaire design, and (c) Procedure for statistical data analysis.

### Population, Sampling, and Collection of Data

The population of the study consists of the people of Pakistan having a maximum age of 40. Forty years maximum age is considered as people before going into their 40’s try to establish long term relationships with other peoples, decide about their occupations, choose a lifestyle and, most importantly, manage their home and family (Bastable, 2017). The unit of analysis is individuals. It is difficult for the researcher to collect data from the whole population, and due to this sampling approach is considered useful for data collection (Vanderstoep and Johnston, 2009). In the present study, the researcher has used a convenient sampling technique that is one of the popular forms of non-probability sampling technique. In the convenient sampling technique, a subset of the population that is conveniently available is used for data collection.

The questionnaire was in the English language. Although English is not a native language in Pakistan but the medium of education in Pakistan is in English. Thus, the people of Pakistan can read and understand English. That is the reason that the researchers used the research questionnaire in the English language. Individuals who were able to read and understand the English language participated in the process of data collection. For the enrollment of the respondents, the researcher tried to contact individuals through personal visits at universities, large organizations, and social media platforms. The selection of these areas was made as large number of potential respondent were present there. The researchers mainly focused on university students as universities provide the opportunity to interact with the students of different gender groups, different educational backgrounds, different social classes, and different geographical locations. The researchers also arranged online sessions with the respondents to interact with them.

Before getting responses to the questionnaire, the researchers provided a presentation to the respondents about the research objective. After the presentation, the researchers distributed the research questionnaires to the respondents who were conveniently available and provided the consent to fill the questionnaire. Respondents included individuals, having age less than 40 years and engaged in any type of earning activity. The researchers have applied this condition to find out the real situation regarding “FWB” when individuals start making their own financial decisions. During the data collection phase, the researchers collected the data from different geographical locations, and tried keep diversity in gender, educational background, other vital indicators. During the data collection process, the researcher maintained the confidentiality of the respondents and followed the ethical guidelines proposed by the APA and the researcher’s community. The results section explains the demographic profile of the respondents.

Krejcie and Morgan (1970), recommended a sample of 384 for 1 million population. Cohen (1992) suggested a 212 minimum sample size for mediation analysis. However, by analyzing the sample size of similar studies, i.e., (Sabri, 2011; Serido et al., 2014; Burcher et al., 2018), 1500 sample size is considered appropriate for the present study. The researcher used a large sample size to generalize the results of the investigation.

### Questionnaire Design

This study is cross-sectional and used validated instruments to collect the data. Before using the questionnaire, the researchers obtained approval from the departmental committees of the university. Important variables that are studied include Financial Well-being, Financial Locus of Control, Financial Socialization, and early childhood consumer experiences.

• Financial Well-Being (FWB): The present study measured FWB by using the In-charge Financial Distress/Financial Well-being scale (IFDWF) developed by Prawitz et al. (2006). This instrument measures levels of stress and well-being, resulting from one’s financial conditions. For each item, responses ranged from 1 to 10. After reversing average individual scores, it ranges from 1 (lowest FWB) to 10 (highest FWB).
• Financial Socialization (FS): In the present study, the researcher has measured Financial Socialization by using the instrument the Financial Social Learning Opportunities “developed by Gudmunson and Danes (2011) and Gutter et al. (2010). The financial, social learning opportunities score was a composite measure based on six dimensions: “discussions with parents,” discussions with peers,” “discussion with teachers,” “observing parents,” “observing peers,” and “observing teachers.” The average score of the financial socialization ranged from 1 (Lowest FS) to 5 (Higher FS).
• Early Childhood Consumer Experience (ECCE): The researcher has measured ECCE by using the instrument developed by Danes (1994). Sample questions were “at what age they became involved in financial activities, which included (a) having their own savings account, and (b) discussing financial matters with parents.” The researchers measured ECCE by taking the average of the above two questions. The average score was from 1 (Low level of ECCE) to 4 (Higher level of ECCE).
• Locus of Control: The researcher measured LOC by modifying the instrument developed by Kesavayuth et al. (2018). The underlying instrument was related to Economics Locus of control, and the researcher modified to use it as a measure of Financial locus of control. It does not affect the meanings and use. There were only a few changes of words. It has seven items measured on 5 points Likert scale ranging from strongly disagree to agree strongly. The researcher used the average of the questions as a measure of financial LOC. The range of average was from 1 (Low Level of Internal LOC) to 5 (High level of Internal LOC).

### Procedure for Statistical Data Analysis

To test the theoretical model, Partial Least Square (PLS) path modeling technique is used. PLS path modeling technique is similar to Structural Equation Modeling (SEM). PLS path modeling technique is a special version of SEM that is Variance based, whereas traditional SEM is a covariance-based technique. These techniques cannot be used alternatively as both techniques have specific uses. SEM is preferred when the objective is to test a theory where all links between variables are pre-defined while the PLS modeling technique is used when finding new links that are not pre-defined (Hair et al., 2017). PLS path modeling technique is applied in the field of management sciences and related disciplines (Hair et al., 2017). The primary objective of the present study was to find the key drivers of FWB among adults that can be achieved adequately by using SmartPLS (Hair et al., 2017).

Furthermore, it is considered the most appropriate technique for the studies having similar objectives (Hair et al., 2017). For the computation of PLS results, we have utilized SMART PLS software. In the PLS path modeling technique, final decisions about the relationship between variables are made based on their coefficients value and *t* values.

## Results

The researchers have collected 1500 responses. After deleting responses having missing values, unengaged responses, and outliers, there were only 1130 valid remaining responses. Table 1 reports the demographics of these 1130 respondents. The analysis results showed that the majority of the respondents were male (72%) having age group 25–30 years (40%). Moreover, the majority of the respondents were single (60%) having a master’s education (73%), and they belong to urban areas of the country. In addition to this, almost 85% of the population was having less than 10-year job experience.

**TABLE 1:** Demographic profile of the respondents.

	Frequency	Percent		Frequency	Percent
**Age**			**Father’s Education**		
<25 Years	328	29%	No formal education	96	8.5%
25–30 Years	456	40.4%	High school and lower	398	35.2%
30–40 Years	346	30.6%	Undergraduate	202	17.9%
**Gender**			Bachelor	270	23.9%
Female	314	27.8%	Master	148	13.1%
Male	816	72.2%	Ph.D. degree	16	1.4%
**Marital status**			**Mother’s education**		
Single	684	60.5%	No formal education	230	20.4%
Married	432	38.2%	High school and lower	456	40.4%
Divorced	14	1.2%	Undergraduate	190	16.8%
**Education**			Bachelor	176	15.6%
High school and lower	4	0.4%	Master	76	6.7%
Undergraduate	38	3.4%	Ph.D. degree	2	0.2%
Bachelor	148	13.1%	**Working experience**		
Master	824	72.9%	<3 Years	498	44.1%
Ph.D. degree	116	10.3%	3–10 Years	474	41.9%
**Academic ability**			Over 10 years	158	14.0%
GPA > 2.50	50	4.4%	**Place of origin**		
GPA 2.5–3.00	244	21.6%	Urban	750	66.4%
GPA 3.00–3.50	464	41.1%	Rural	380	33.6%
GPA < 3.50	372	32.9%			

*Source: Researcher.*

Hair et al. (2017) recommended that, before applying a path modeling technique, the researcher should check data for normality and missing values. To compute path modeling technique, Hair et al. (2017) proposed a two-step process, that is:

Evaluation of the measurement model
Evaluation of the structural model

To evaluate the measurement model, researchers need to determine reliability and validity (concerning the content, convergent, and discriminant) of the instruments (Hair et al., 2017).

### Reliability of the Instrument

The present study assessed the reliability statistics by looking into the outer factor loading of each construct individually (Hair et al., 2017). Outer factor loading above 0.4 is considered acceptable (Field, 2017; Hair et al., 2017). Table 2 shows the outer factor loading of each of the latent variables of the present study. Table 2 shows that all values of the out loading are considerably up from 0.4, indicating that all variables of the current research meet the individual item reliability criteria.

**TABLE 2:** Factor loadings, average variance extracted, and composite reliability.

Latent constructs and indicators	Standardized loadings	AVE	CR	Cronbach’s alpha
Financial well-being		0.57^*a*^	0.913	0.891
FWB_1	0.703			
FWB_2	0.771			
FWB_3	0.804			
FWB_4	0.803			
FWB_5	0.706			
FWB_6	0.69			
FWB_7	0.689			
FWB_8	0.854			
*Financial Socialization – Teachers*		0.415^*a*^	0.876	0.847
FSDT_1	0.852			
FSDT_2	0.81			
FSDT_3	0.864			
FSDT_4	0.865			
FSDT_5	0.826			
FSOT_1	0.838			
FSOT_2	0.828			
FSOT_3	0.864			
FSOT_4	0.859			
FSOT_5	0.802			
*Financial Socialization – Parents*		0.415^*a*^	0.876	0.847
FSDP_1	0.628			
FSDP_2	0.627			
FSDP_3	0.539			
FSDP_4	0.638			
FSDP_5	0.597			
FSOP_1	0.729			
FSOP_2	0.67			
FSOP_3	0.696			
FSOP_4	0.676			
FSOP_5	0.622			
*Financial Socialization – Peers*		0.573^*a*^	0.931	0.918
FSDPE_1	0.756			
FSDPE_2	0.715			
FSDPE_3	0.751			
FSDPE_4	0.809			
FSDPE_5	0.737			
FSOPE_1	0.725			
FSOPE_2	0.778			
FSOPE_3	0.748			
FSOPE_4	0.789			
FSOPE_5	0.759			
*Early Childhood Consumer Experiences*		0.899^*a*^	0.947	0.89
ECCE_1	0.96			
ECCE_2	0.936			
*Locus of Control*		0.453^*a*^	0.849	0.793
LOC_1	0.828			
LOC_2	0.655			
LOC_3	0.585			
LOC_4	0.513			
LOC_5	0.524			
LOC_6	0.683			
LOC_7	0.843			

*^*a*^ = *p* < 0.05. Source: Researcher.*

### Internal Consistency Reliability

Internal consistency reliability is a measure of how well an instrument is measuring what the researcher wants to measure by using it. A composite reliability coefficient measures it, and a threshold value of 0.7 or above is considered appropriate (Hair et al., 2017). Similarly, Cronbach’s Alpha also performs the reliability of the instruments (Field, 2017). A construct having a value of 0.6 or above is considered appropriate. Table 2 reports the composite reliability coefficients of the latent variables of the study. It shows that all values are above 0.7, showing the composite reliability of the measures used in the study. Cronbach’s Alpha in Table 2 also confirms that all measures used in the present study have internal consistency.

### Convergent Validity

Convergent Validity or indicator reliability is an extent to which a measure correlates positively with the alternative means of the same construct (Hair et al., 2017). Average Variance Extracted (AVE) is used to test the convergent validity (Hair et al., 2017). AVE value should be at least 0.5 or more to indicate convergent validity (Chin, 2010). Table 2 presents the Average Variance Extracted from all the constructs. All values of AVE are above 0.5 except for Financial locus of control and Financial Socialization through parents. *P* values of the AVE show that all values are statistically significant to indicate that the study has adequate convergent validity.

#### Discriminant Validity

Discriminant validity indicates the extent to which a given construct differs from other constructs. The researcher applied Heterotrait-monotrait (HTMT) ratio to assess discriminant validity. The value of HTMT should be less than 0.9 to indicate discriminant validity (Henseler et al., 2015). All the values of HTMT shown in Table 3 are less than 0.9; that is apparent that all study constructs have discriminant validity.

**TABLE 3:** Discriminant validity [Heterotrait-monotrait ratio (HTMT)].

	ECCE	FSP	FSPE	FST	FWB	LOS
ECCE						
FSP	0.201					
FSPE	0.134	0.651				
FST	0.125	0.626	0.713			
FWB	0.347	0.197	0.11	0.135		
LOC	0.101	0.421	0.333	0.371	0.436	

*Source: Researcher.*

### Structural Model Assessment

**FIGURE 2:**
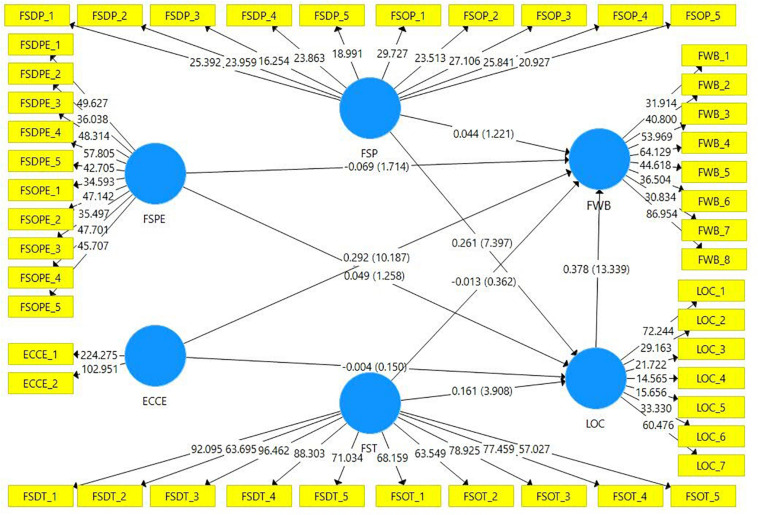
Regression coefficients.

In the present study, to find the path coefficients, the author used the methodology proposed by Hair et al. (2017). The researchers used the bootstrapping resampling procedure (10000 subsamples of the original sample) to obtain the significant values of the indicators. Figure 2 and Table 4 shows the full estimates of the structural model along with statistics about mediating variables for the financial well-being. Originally H_1_ proposed that Financial Socialization through Parents (FSP), financial socialization through Peers (FSPE), and financial socialization through teachers (FST) affects the FWB. Table 4 and Figure 2 have revealed that FSP (*b* = *0.044, t* = *1.221, p* > *0.1*) have no effect on the FWB. Statistics related to FSPE (*b* = *−0.069, t* = *1.703, p* < *0.1)* showed that FSPE has significant effect on the FWB of the adults. The negative sign of the coefficient showed that the effect of FSPE on the FWB is negative but statistically significant. The results also revealed that FST (*b* = *−0.013, t* = *0.358, p* > *0.1)* has no effect on the FWB of the adults in Pakistan. Summing up all the statistics related to H1, we can conclude that FS has a partial effect on the FWB, as only FSPE was relevant to the FWB. The results of the study revealed that among all FS agents, only socialization with peers has a significant effect on FWB. Hence, the researcher found partial support for the H1.

**TABLE 4:** Direct, indirect and total effect of the relationships.

	Direct effect	Indirect effect^1^	Total effect
ECCE − > LOC	*−*0.004		*−*0.004
	(0.151)		(0.151)
ECCE − > FWB	0.292^*c*^	*−*0.002	0.291^*c*^
	(10.203)	(0.15)	(9.731)
LOC − > FWB	0.378^*c*^		0.378^*c*^
	(13.382)		(13.382)
FSP − > LOC	0.261^*c*^		0.261^*c*^
	(7.225)		(7.225)
FSP − > FWB	0.044	0.098^*c*^	0.143^*c*^
	(1.221)	(6.437)	(3.721)
FSPE − > LOC	0.049		0.049
	(1.267)		(1.267)
FSPE − > FWB	*−*0.069^*a*^	0.019	*−*0.05
	(1.703)	(1.255)	(1.166)
FST − > LOC	0.161^*c*^		0.161^*c*^
	(3.922)		(3.922)
FST − > FWB	*−*0.013	0.061^*c*^	0.048
	(0.358)	(3.81)	(1.237)

*^1^LOCUS of control (LOC) is a mediating variable. Values in the parenthesis are *t* values. ^*a*^*p* < 0.1. ^*b*^*p* < 0.05. ^*c*^*p* < 0.001. Source: Researcher.*

H_2_ was the early childhood consumer experience (ECCE) effect on FWB. Figure 2 and Table 4 shows that ECCE (*b* = *0.292, t* = *10.203, p* < *0.001)* have significant effect on the FWB. The results indicate that with the increase in the value of early childhood consumer experiences, the FWB in adulthood will also increase. Hence, the results of the study supported H_2_ that ECCE has a significant effect on FWB.

H_3_ was that Financial Locus of Control (LOC) mediates the relationship between FS through Parents, Peers, Teachers, and FWB. Figure 2 and Table 4 show the indirect effect from independent to dependent variable. The coefficient of indirect effect of FSP (*b* = *0.098, t* = *6.437, p* < *0.001)*. The statistics related to FSP shows that LOC mediates the effect of FSP on the FWB as the *t* value of the indirect effect is significant. For FST (*b* = *0.061, t* = *3.81, p* < *0.001)* shows the evidence of the mediating effect of LOC between FST and FWB. The coefficient of indirect effect of FSPE (*b* = *0.019, t* = *1.255, p* > *0.1)* shows no mediating effect of LOC between FSPE and FWB. Hence, the researchers will partially support H3 as LOC works as a mediator only for FSP and FST.

H4 was that LOC mediates the relationship between ECCE and FWB. The indirect effect from Table 4 and Figure 2 reveals the coefficient of ECCE (*b* = *−*0.002, *t* = 0.150, *p* > 0.1) confirms that LOC does not mediate the relationship between ECCE and FWB.

## Discussion and Conclusion

The present study is an attempt to develop and test a conceptual model of FWB based on FS and ECCE. The present study is among the few pioneer studies that try to explore the link between FS, ECCE, and FWB among the adults of Pakistan. The results of the research provided support for our theoretical framework. The theory of consumer socialization, the theory of life-span development, and the theory of planned behaviors provided the basis for the theoretical model of the study.

The results of the present study partially supported the notation that through the financial socialization (FS) process as well as early childhood consumer experiences, adults can improve their FWB. Contrary to the previous studies (Shim et al., 2009a,b; Sabri and MacDonald, 2010; Serido et al., 2010; Sabri et al., 2012), our study showed that FS through peers exhibited a negative effect on the FWB of the individuals. That shows the FWB of the adults reduces who discusses and observes the financial behaviors of the peers. Usually, people who try to follow and observe the financial pattern of their peers often face difficulties in managing their financials. These difficulties arise due to the lack of confidence and lack of peer support at the time of stress. Schofield and Hotulainen (2004) showed that adults who receive no specific support from peers perform better than their control group, where individuals get peer support. Beshears et al. (2015) also showed the negative effect of peer information support on the savings. Based on this, we can say that the results of the present study are in line with (Schofield and Hotulainen, 2004; Beshears et al., 2015) that a high level of association with peers impaired decision-making process that results in a low level of financial well-being among the adults.

The results also revealed that financial socialization through teachers and parents have no direct effect on the FWB of adults. Parents are important socialization agents that help in improving financial decision making among adults (Drever et al., 2015). There are two possible explanations for this. The first is the lack of knowledge among the parents. Table 1 reports that only 14% of parents had a Master’s degree or above qualification. If parents have limited knowledge about the financials, then they cannot guide their children. In Pakistan, financial literacy programs were started in the early 1990s. That is the reason that parents have a low level of financial proficiency. Furthermore, they are unable to discuss it with their children. However, parents helped children in building locus of control that results in higher level FWB. Our study also supports this relationship.

Early childhood consumer experiences (ECCE) were having a direct effect on the FWB of adults. Consistent with Sabri et al. (2012), the present study supports the hypothesis that the adults having bank accounts or other saving mechanisms in childhood have a positive effect on the financial decisions made in adulthood. ECCE provides confidence to the adults that they can manage their finance in a batter way. Proper management of financials helps in achieving FWB during adulthood. Peng et al. (2007) also argued that experiences help more in making sound investments and saving decisions than financial education.

The results of the study revealed that locus of control mediates the relationship between FS through parents, teachers, and FWB, which confirms that FWB for the adults will be higher for those who discuss and observe financial behaviors with parents and teachers and have belief in their own. Individuals having an internal LOC are more likely to work hard to achieve their goals. Discussions with the parents and teachers on financial matters and observation of parents and teachers give confidence as well as knowledge to manage their finances. These improvements, along with internal LOC, results in better financial decisions. It ultimately leads to improvement in the FWB among the adults. LOC does not work for socialization through Peer and Early Childhood consumer experiences. The result of the present study is confirmatory (Prawitz and Cohart, 2016).

The financial decisions of the individuals are likely to be influenced by those with whom they interact. Peoples in their adulthoods usually spend more time with their parents and teachers and follow their decision patterns. The findings of the study supported this argument. That means that parents and teachers are in a better position to guide adults about financial decisions. For the results, we found support from the study (Oaten and Cheng, 2007). The discussion with the parents and teachers enables self- governing skills in individuals. It enables adults to face financial difficulties more confidently due to the confidence they gained by discussing and observing their parents on financial matters. In other words, due to the belief that they can control what is happening around, adults can handle the financial problems they face and can achieve financial well-being. The results suggested that individuals who discuss and observe the financial decision making patterns of their parents, as well as have LOC, improve their FWB.

Based on the result of the present study, we can say that there should be awareness programs for the teachers as well as parents and children as well. These programs should highlight the importance of sharing and practicing positive financial practices at homes and schools. In addition to this, these programs should be part of early education at school’s levels to motivate children to save and invest in their childhood. The contents should focus on experience-based, practical learning rather than the theoretical views about the management of financials. Through all these practices, children will learn about money-related activities and improve their financial well-being in adulthoods. The other important thing is that individuals’ parents, teachers, and peers should give confidence to the children that they can control things on their own. Although it is difficult for someone to develop it still, adults in their childhood were in a better position to learn new things and boost their confidence level. Parents and teachers can play a critical role in giving confidence to the children. That will help them to make in making better financial decisions and adjust themselves to the changing environment.

Although the present study has some important implications for the individuals, parents, teachers, society, and government, it has some limitations. In this study, self-administrated questionnaires were used to get data from the population-based on their judgments. Multiple sources of information should be utilized to collect more accurate data. By using the cross-sectional data, we cannot adequately explore the causal relationship among variables. Longitudinal data are required to test the causal connection. Some other financial behaviors should be considered while developing a model of FWB.

## Data Availability Statement

The datasets used in this study is not readily available because this paper is a part of the Ph.D. thesis of SU. Before of Ph.D. requirements, the data cannot be shared with anyone. However, individuals can forward their requests to access the datasets to Saifullah271@yahoo.com.

## Ethics Statement

Ethical review and approval was not required for the study on human participants in accordance with the local legislation and institutional requirements. The patients/participants provided their written informed consent to participate in this study.

## Author Contributions

SU was involved in all the steps and procedures followed in this study, and the present study is a part of his Ph.D. thesis. SU was involved in conceptualization, reviewing the literature, finalizing research methodology, data collection and analysis, writing, and reviewing the original draft. KY was Ph.D. supervisor of SU at Jiangsu University of Zhenjiang. KY supervised this project and supported in all the steps followed in this study and played a supporting role in the conceptualization, reviewing the draft. Both authors contributed to the article and approved the submitted version.

## Conflict of Interest

The authors declare that the research was conducted in the absence of any commercial or financial relationships that could be construed as a potential conflict of interest.
